# Human Milk Oligosaccharides Variation in Gestational Diabetes Mellitus Mothers

**DOI:** 10.3390/nu15061441

**Published:** 2023-03-16

**Authors:** Yuqi Dou, Yuanli Luo, Yan Xing, Hui Liu, Botian Chen, Liye Zhu, Defu Ma, Jing Zhu

**Affiliations:** 1 Department of Social Medicine and Health Education, School of Public Health, Peking University Health Science Center, Beijing 100191, China; douyuqi@bjmu.edu.cn (Y.D.);; 2 School of Public Health, Sichuan University, Chengdu 610041, China; 3 Department of Pediatrics, Peking University Third Hospital, Beijing 100191, China; 4 Obstetrics Department, Maternal and Child Hospital of Haidian District, Beijing 100080, China; 5 Institute of Biotechnology and Health, Beijing Academy of Science and Technology, Beijing 100089, China

**Keywords:** gestational diabetes mellitus, human milk oligosaccharides, phenotype, lactation, infant growth

## Abstract

Gestational diabetes mellitus (GDM) is a common disease of pregnancy, but with very limited knowledge of its impact on human milk oligosaccharides (HMOs) in breast milk. This study aimed to explore the lactational changes in the concentration of HMOs in exclusively breastfeeding GDM mothers and the differences between GDM and healthy mothers. A total of 22 mothers (11 GDM mothers vs. 11 healthy mothers) and their offspring were enrolled in the study and the levels of 14 HMOs were measured in colostrum, transitional milk, and mature milk. Most of the HMOs showed a significant temporal trend with decreasing levels over lactation; however, there were some exceptions for 2′-Fucosyllactose (2′-FL), 3-Fucosyllactose (3-FL), Lacto-N-fucopentaose II (LNFP-II), and Lacto-N-fucopentaose III (LNFP-III). Lacto-N-neotetraose (LNnT) was significantly higher in GDM mothers in all time points and its concentrations in colostrum and transitional milk were correlated positively with the infant’s weight-for-age Z-score at six months postnatal in the GDM group. Significant group differences were also found in LNFP-II, 3′-Sialyllactose (3′-SL), and Disialyllacto-N-tetraose (DSLNT) but not in all lactational periods. The role of differently expressed HMOs in GDM needs to be further explored by follow-up studies.

## 1. Introduction

Breast milk contains all the nutrients needed to support infant growth and development; therefore, it is the ideal source of nutrition for infants in the first six months of life and is recognized as the gold standard for feeding babies [1]. There have been many studies showing both short- and long-term health benefits of human milk for both the mother and the offspring [2,3,4,5].

Human milk oligosaccharides (HMOs) are the third largest solid component of human milk after lactose and lipid, which are water-soluble oligosaccharides secreted by the mother’s mammary glands during pregnancy and lactation [6]. HMOs are composed of 3–23 monosaccharide molecules including D-glucose (Glc), D-galactose (Gal), N-acetyl-glucosamine (GlcNAc), L-fucose (Fuc), and sialic acid (Sia) [7], of which N-acetylneuraminic acid (Neu5Ac) is the most predominant form of sialic acid [8]. Based on the terminal monosaccharide, HMOs can be classified into four types: (1) fucosylated sialylated type, (2) non-fucosylated non-sialylated type, (3) fucosylated non-sialylated type, and (4) non-fucosylated sialylated type. Approximately 200 oligosaccharide structures have been identified in human milk [6,9,10]. HMOs have shown the ability to reduce infection, maintain intestinal micro-ecological balance, and be involved in immune regulation [2,11,12,13,14,15]. The concentration of HMOs is influenced by a variety of factors, including genetics (e.g., secretor and Lewis genes), geographic location, gestational age, parity, lactation stage, maternal age, weight, body mass index (BMI), mode of delivery, maternal diet, race, socioeconomic status, other environmental factors (e.g., seasonality), infant gestational age, and infant sex [16,17,18,19].

Gestational diabetes mellitus (GDM) is a common disease of pregnancy in which diabetes mellitus first occurs during pregnancy. The prevalence of GDM in China is about 18–20% [20]. GDM is associated with maternal metabolism and altered intestinal flora [21]. It has been found that GDM can alter the composition of maternal blood [22,23] and placenta [24], and therefore may also affect the composition of human milk. In addition, it may also alter the activity of glycosyltransferases and glycosidases [25], thereby altering the glycopolymers in human milk, such as HMOs. HMOs are already present in the maternal circulation during pregnancy and the increase of some sialylated HMOs under GDM was found, which may be linked to the higher sialylation status caused by chronic inflammation [26,27]. In a group of overweight and obese women, serum 3′-Sialyllactose (3′-SL) levels were found to be higher in early pregnancy in women who would develop GDM in the future [27]. In addition to changes in maternal blood HMOs, Hirschmugl et al. [28] demonstrated that the profile of HMOs in neonatal cord blood was similar to that in maternal blood at delivery, and Hoch et al. [29] also found GDM-related alterations in maternal HMOs in the neonatal circulation, with higher 3′-SL in GDM cord blood. Moreover, only two studies examined the differences in the levels of HMOs in human milk between lactating GDM mothers and healthy mothers, but the findings were not consistent. In 2013, Smilowitz et al. [30] found no difference in mean abundance of HMOs in the transitional milk (two weeks postpartum) of women with and without gestational diabetes. Later in 2022, Wang et al. [31] analyzed 13 HMOs in colostrum and demonstrated that GDM colostrum had lower levels of sialylated oligosaccharides, especially 3′-SL, compared to healthy secretor mothers. The findings of HMOs in breast milk were inconsistent. Moreover, how HMOs change over lactational time in GDM mothers’ milk and the difference from healthy mothers’ milk at the same time remains unclear. Currently, there are also many studies investigating the association between the composition of HMOs in breast milk and infant growth and development indicators, but the conclusions are not yet consistent [32,33,34,35].

In this study, 14 HMOs in colostrum, transitional milk, and mature milk from parturients with and without GDM were quantified by an ion chromatography-based approach and the lactational changes of HMOs were compared between GDM and healthy mothers. Moreover, the possible factors influencing the content of HMOs and the correlation with infants’ health and growth outcomes (disease status, bowel movements, infant length, head circumference, and weight) were explored.

## 2. Materials and Methods

### 2.1. Ethical Review and Project Registration

The study was approved by the Medical Science Research Ethics Committee of Peking University Third Hospital (Ethics Committee approval number: IRB00006761-M2021062) and registered (Registration number: ChiCTR2100045411).

### 2.2. Subjects and Sample Collection

#### 2.2.1. Study Subjects

This study is part of a prospective cohort study. In the cohort study, mother–infant pairs were enrolled at Peking University Third Hospital and Beijing Haidian Maternal and Child Health Hospital from June 2021 to April 2022. GDM was screened during each participant’s routine 24- to 28-week prepartum clinical visit using a 75 g, 2 h oral glucose tolerance test (OGTT). Participants whose fasting glucose exceeded 5.1 mmol/L or 1 h OGTT >10.0 mmol/L or 2 h OGTT >8.5 mmol/L were diagnosed as GDM [36]. Additionally, these mothers do not suffer from other pregnancy disorders besides GDM, such as gestational hypertension, hypothyroidism, and polycystic ovary syndrome. We included women with GDM who received only nutritional and exercise therapy to achieve glycemic control goals, without treatment with insulin and oral hypoglycemic agents. Good glycemic control was defined according to the recommended glycemic control goal by the American Diabetes Association (ADA) [37] and the American College of Obstetricians and Gynecologists (ACOG) [38], which is fasting and pre-meal glucose levels <95 mg/dL (5.3 mmol/L) and 2 h postprandial glucose level <120 mg/dL (6.7 mmol/L).

Other inclusion criteria hold the same for both GDM and healthy groups. Mothers were included when aged 18–45 years and giving birth to a healthy term single baby. Mothers were excluded if they had a history of type 1 or type 2 diabetes or a history of abnormal glucose tolerance before pregnancy. Both groups were willing to exclusively breastfeed. We conducted a survey of feeding patterns at 42 days postpartum to find out the percentage of exclusive breastfeeding.

In addition to the requirement of subject enrollment, for this study, the subjects were further selected to minimize the differences of possible confounders between GDM and healthy participants, such as maternal age, pre-pregnancy BMI, gestational age, parity, mode of delivery, and gender of the infant [16,17,18]. Pre-pregnancy BMI (weight (kg)/height (m)^2^) was classified as follows: underweight (<18.5 kg/m^2^), normal weight (18.5–24.9 kg/m^2^), overweight (25.0–29.9 kg/m^2^), and obese (≥30.0 kg/m^2^). All mothers selected in this study had a normal weight according to pre-pregnancy BMI. Therefore, 11 GDM and 11 healthy mothers and their offspring were selected in this study.

#### 2.2.2. Determination of Milk Type

The presence or absence of α1,2-fucosylated and α1,4-fucosylated HMOs could be utilized to classify the breast milk samples into different milk types. By adapting the classification method of Wang et al. [17], the level of 2′-FL was used to determine secretor (Se) type, while LNFP-II for Lewis (Le) type, as they represented the presence of α1,2-fucosylated and α1,4-fucosylated HMOs, respectively. Lactating mothers containing α1,2-fucosylated structures were classified as secretor-positive (Se^+^) and others were classified as non-secretor (Se^−^); for lactating mothers containing α1,4-fucosylated HMOs, they were classified as Lewis-positive (Le^+^) and others were Lewis-negative (Le^−^). Therefore, lactating mothers could be classified into 4 groups, namely Se^+^Le^+^, Se^+^Le^−^, Se^−^Le^+^, and Se^−^Le^−^.

#### 2.2.3. Milk Sample Collection and Storage

Milk samples were collected from different stages of postpartum lactation, including within 7 days (colostrum), 12–14 days (transitional milk), and 42 days (mature milk) postpartum. Human milk sampling was standardized for all participants. In short, after cleaning the right breast which was not used to feed the infant at least 4 h before collection, whole breast milk samples were collected between 9:00 a.m. and 11:00 a.m. using a breast pump, followed by mixing and transferring into 50 mL sterilized centrifuge tubes. Samples were immediately frozen at −20 °C, transferred in a cold chain to the lab within 6 h, aliquoted on ice when received, and stored at −80 °C until thawed for analysis.

#### 2.2.4. Data Collection and Definition of Variables

For mothers, basic information included sociodemographic characteristics (age, occupation, and income), obstetric characteristics (pre-pregnancy weight, gestational age, and mode of delivery), and self-reported allergy history were collected. The information was obtained from records in the medical record system and questionnaires. Gestational weight gain (GWG) was calculated by the difference between the weight at the pre-delivery examination and the pre-pregnancy weight. Based on the history of delivery, the mothers were divided into primiparous and multiparous.

For infants, general demographic information was recorded using only measurements provided by community health centers or hospitals, such as gender, weight, length, and head circumference. We used the World Health Organization Anthro software anthropometric calculator [39] to calculate children’s length-for-age Z-score (LAZ), weight-for-age Z-score (WAZ), and BMI-for-age Z-score (BMIZ), respectively. The World Health Organization 2006 criteria [40] were used to determine the Z-score cut-offs for growth status of children under 5 years of age: BMIZ at age >3 for obesity, >2 for overweight, <−2 for wasting, and <−3 for severe wasting; WAZ <−2 for low weight and <−3 for severe low weight; and LAZ <−2 for growth retardation and <−3 for severe growth retardation. In addition to this, infant health conditions reported by parents were also collected, including infant eczema, respiratory and digestive disorders, as well as feeding and bowel movements.

### 2.3. Quantification of HMOs

The 14 HMOs standards were purchased from GLYCOSCI (ZZBIO, Shanghai, China), which were Lacto-N-tetracose (LNT) (Purity: 95%), lacto-N-neotetraose (LNnT) (Purity: 99%), 2′-Fucosyllactose (2′-FL) (Purity: 91.3%), 3-Fucosyllactose (3-FL) (Purity: 91%), Lacto-N-fucopentaose I (LNFP-I) (Purity: 95%), Lacto-N-fucopentaose II (LNFP-II) (Purity: 95%), Lacto-N-fucopentaose III (LNFP-III) (Purity: 95%), Lacto-N-difucosylhexaose I (LNDFH-I) (Purity: 95%), Sialyllacto-N-tetraose a (LST a) (Purity: 95%), Sialyllacto-N-tetraose b (LST b) (Purity: 95%), Sialyllacto-N-tetraose c (LST c) (Purity: 95%), Disialyllacto-N-tetraose (DSLNT) (Purity: 98%), 3′-SL (Purity: 98%), and 6′-Sialyllactose (6′-SL) (Purity: 98.7%). We prepared the standard reserve solution of 14 HMOs by accurately weighing each HMOs standard, dissolving them in pure water, and fixing the volume in volumetric flasks. Next, we diluted the standard reserve solution to obtain a series of standard working solutions with different concentrations.

Furthermore, we thawed the human milk sample at 4 °C and took 0.2 mL of it to mix with 1.8 mL of warm water (60 °C), and then centrifuged the mixture at 8000 r/min at room temperature for 10 min. Next, we took the supernatant and filtered it through a 0.45 µm microporous membrane (Tianjin Jinteng Company, Tianjin, China) before ion chromatography analysis.

The HMOs were separated by an ion chromatograph LC System (ICS-3000 ion chromatograph, Thermo Fisher, San Jose, CA, USA) equipped with a DIONEX CarboPacTM PA1 protective column (4 mm × 50 mm, 10 µm, Thermo Fisher Scientific, Waltham, MA, USA) and a DIONEX CarboPacTM PA1 analytical column (4 mm × 50 mm, 10 µm, Thermo Fisher Scientific). The column temperature was 30 °C and the flow rate was 1.0 mL/min. Mobile-phase solvents A, B, and C consisted of deionized water, 200 mM sodium hydroxide, and 150 mM sodium hydroxide + 500 mM sodium acetate, respectively. A pulse amperometric detector (Thermo Fisher Scientific, San Jose, CA, USA) was used for signal detection.

The series of standard working solutions of HMOs were injected into the ion chromatograph separately. The standard curve was plotted with the concentration of the standard working solution as the horizontal coordinate and the peak area as the vertical coordinate. The milk sample was analyzed by the same parameter as the standard working solution. The peak of each HMO was determined by retention time and the peak area was measured. The concentration of HMOs in the human milk sample was calculated by the standard curve.

### 2.4. Statistical Analysis Methods

Before analysis, the distribution of continuous variables was assessed by histograms, skewness and kurtosis measures, and Shapiro–Wilk tests. Means and standard deviations (normal distribution measurement data), median and interquartile range (skewed distribution measurement data), and composition ratios (enumeration data) were used to describe maternal and infant sociodemographic, anthropometric characteristics, and HMOs concentration.

To assess differences between mothers with and without GDM, we used two independent samples *t*-test for group comparisons when the measurement data were normally distributed, and Mann–Whitney U in non-parametric tests was used when the measurement data were skewed. When the data type was categorical data, Fisher’s exact test was used to compare the difference between the two groups. Generalized estimating equation analysis (GEE) was used to study changes and differences in HMOs between the healthy and GDM groups at different stages of lactation. *p* < 0.05 was the level of significance. If there was no time and group interaction, the interaction term was removed, and the main effects analysis continued. When the time main effects were statistically different, one-way generalized estimating equation analysis was performed within each of the two groups. When the group main effects were statistically significant, grouped univariate analyses were performed for each time point. If there was an interaction between group and time, separate effects analysis was performed for time and group. Moreover, we used GEE to explore whether there were differences in indicators of infant growth and development in the two groups.

Exploratory analyses were performed using Spearman’s rank correlation analysis to investigate the correlation between maternal characteristics (age, gestational age, pre-pregnancy BMI, gestational weight gain, mode of delivery, and allergic diseases) and infant gender and individual HMO concentrations at different time points. For these analyses, a significance level of *p* < 0.05 was used, with correlation levels interpreted as 0 to 0.19—very weak correlation, 0.20 to 0.39—weak correlation, 0.40~0.69—moderate correlation, 0.70~0.89—strong correlation, and 0.90~1.00—very strong correlation.

A stacked bar chart plot was used to represent the relative concentrations of HMOs. Histograms were used to graphically demonstrate the variation of HMO concentrations over time in the two groups.

## 3. Results

### 3.1. Basic Information on Maternity and Infancy of Both Groups

A total of 22 mother–infant pairs (11 pairs each in the GDM and healthy groups) enrolled in the study, with mothers aged between 28 and 40 years and delivery gestational weeks between 38^+2^ and 41^+3^ weeks. In each group, 9 out of 11 (81.8%) were primiparous, and 8 (72.7%) had a vaginal delivery. The mothers with or without GDM showed no difference in their basic information (Supplementary Table S1). For GDM mothers, they were diagnosed as GDM at 25.26 ± 0.68 weeks, and the OGTT test results were 5.14 ± 0.28 mmol/L on fasting, 9.83 ± 1.67 mmol/L at 1 h, and 8.23 ± 1.24 mmol/L at 2 h. According to the recommended values of glycemic control goals, three women with GDM had good glycemic control and seven had poor glycemic control in this study. Moreover, one woman lacked prenatal visit data. The blood glucose level of the GDM group at the follow-up is shown in Supplementary Table S2.

For infants, no difference was noticed in the two groups when they were born, with birth weight ranging from 2790 g to 4060 g, birth length ranging from 48 cm to 52 cm, and birth head circumference ranging from 32 cm to 35 cm and five males in each group (Supplementary Table S1). For infants of GDM mothers, the neonatal blood glucose at birth was 3.81 ± 1.52 mmol/L (Supplementary Table S2).

### 3.2. Milk Type

Based on the level of 2′-FL and LNFP-II, the mothers involved in this study were categorized into four phenotypes, namely Se^+^Le^+^, Se^+^Le^−^, Se^−^Le^+^, and Se^−^Le^−^. The distribution of the four milk types is shown in Supplementary Table S3**.** In our study, most of the mothers belonged to Se^+^Le^+^ phenotype, which accounted for 68.2% of 22 mothers, and the rest were Se^-^Le^+^ phenotype. None of the mothers were Se^+^Le^−^ or Se^−^Le^−^. In GDM mothers, seven (63.6%) were Se^+^Le^+^ and four (36.4%) were Se^−^Le^+^, while in healthy mothers, the numbers were eight (72.7%) and three (27.3%), respectively.

Since the milk type has an unignorable effect on the concentration of HMOs, the comparison of GDM with healthy mothers should be within the same milk type. As Se^+^Le^+^ is the major phenotype of subjects in this study, the following analysis mainly focused on it. The basic information of the phenotype Se^+^Le^+^ of GDM and healthy subjects showed no significant differences. Detailed information is shown in Table 1.

### 3.3. Changes of Total HMOs in GDM and Healthy Se^+^Le^+^ Mothers during Lactation

**Figure 1:**
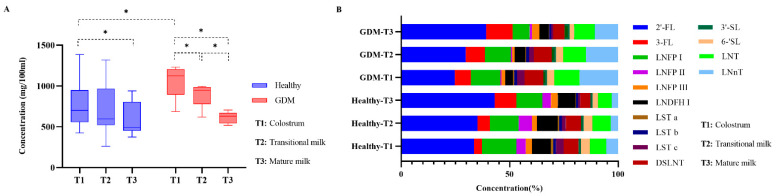
(**A**) Total content of HMOs (only Se^+^Le^+^ mothers); the bracket showed the two conditions to be compared, and * indicated the difference was statistically significant. (**B**) Relative abundance of individual HMOs (only Se^+^Le^+^ mothers). The 14 individual HMOs were color-coded.

The total level of HMOs was represented by the sum of 14 HMOs. In Se^+^Le^+^ mothers, the total level of HMOs in colostrum, transitional milk, and mature milk were 8.90 ± 2.87 g/L, 7.78 ± 2.69 g/L, and 5.98 ± 1.55 g/L, respectively. There was a significant downward trend in total HMOs over lactation in all groups (Figure 1A). In colostrum, the total of HMOs was significantly higher in GDM than healthy mothers (*p* = 0.030); however, no significant difference was observed for transitional milk and mature milk (Figure 1A).

### 3.4. Changes of Individual HMOs in GDM and Healthy Se^+^Le^+^ Mothers during Lactation

Stacked histograms were used to represent the relative abundances of HMOs (Figure 1B). The level of 2′-FL was the most abundant HMO in colostrum (33.75% in healthy group and 24.45% in GDM group), transitional milk (33.09% in healthy group and 30.62% in GDM group), and mature milk (43.54% in healthy group and 38.11% in GDM group). In the healthy group, 3′-SL was the lowest HMO in colostrum (1.00%) and transitional milk (1.16%) and LST a (0.34%) was the lowest in mature milk; in the GDM group, LNFP-II was the lowest HMO in colostrum (0.46%) and LST a was the lowest in transitional milk (0.45%) and mature milk (0.29%).

Time trend analysis within each group showed that non-fucosylated non-sialylated HMOs such as LNT and LNnT decreased with milk maturation in both groups; for fucosylated non-sialylated HMOs, LNFP-I in the GDM group and LNDFH-I in the healthy group decreased significantly from colostrum to mature milk. LNFP-III in both groups and LNFP-I in the healthy group underwent a fluctuation that had the lowest value in the transitional milk. LNDFH-I in GDM group reached the peak in the transitional milk. For non-fucosylated sialylated HMOs, 6′-SL, LST a, LST b, and LST c in both groups and 3′-SL and DSLNT in the GDM group decreased significantly from colostrum to mature milk. DSLNT in the healthy group reached the peak in the transitional milk (Table 2).

For the same lactational period, LNnT continued to show a higher concentration in GDM mothers’ milk than in healthy mothers over lactation (*p* = 0.002, 0.005, and 0.007 in colostrum, transitional, and mature milk, respectively). LNFP-II in colostrum (*p* = 0.010) and transitional milk (*p* = 0.038) also differed significantly between the two groups, with higher concentrations in the healthy group than in the GDM group. There were significant differences of 3′-SL in colostrum (*p* = 0.040) and mature milk (*p* = 0.040) between the two groups, with higher levels in the GDM group than in the healthy group. Between-group differences were observed in DSLNT only in colostrum (*p* = 0.014), with the GDM group being higher than the healthy group (Table 2).

### 3.5. Relations between Basic Maternal and Infant Information and HMO Concentrations

**Figure 2:**
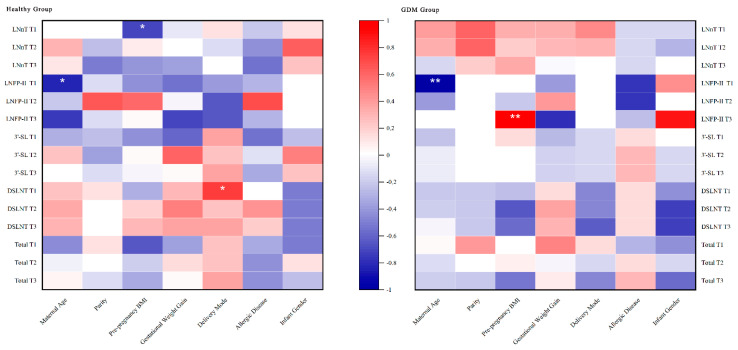
Spearman rank correlations between basic maternal and infant information and individual HMO concentrations in the two groups of the Se^+^Le^+^ phenotype. T1, T2, and T3 represent colostrum (within 7 days), transitional milk (12–14 days), and mature milk (42 days), respectively. ** represents the correlation is significant at the 0.01 level (two-tailed). * represents the correlation is significant at the 0.05 level (two-tailed).

The basic conditions of mother and infant were analyzed to discover other factors related to the concentration of HMOs. Spearman correlation analysis showed that maternal age was negatively correlated with LNFP-II in colostrum in the healthy and GDM groups; pre-pregnancy BMI showed a significant negative correlation with LNnT in colostrum in the healthy group and a significant positive correlation with LNFP-II in mature milk in the GDM group; and delivery mode showed a positive correlation with DSLNT in colostrum in the healthy group. No association was found between parity, GWG, maternal presence of allergic diseases, and infant gender with HMOs levels (Figure 2).

### 3.6. Infant Growth and Development in Relation to HMO Concentration

**Figure 3:**
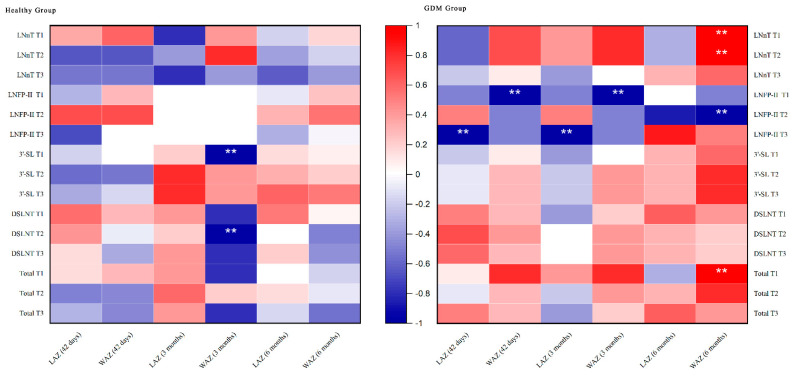
Spearman rank correlations between infant growth indicators and individual HMO concentrations in the two groups of the secretory type. T1, T2, and T3 represent colostrum (within 7 days), transitional milk (12–14 days), and mature milk (42 days), respectively; ** represents the correlation is significant at the 0.01 level (two-tailed).

Data on infant growth at 42 days, 3 months, and 6 months after birth showed that no significant group differences were observed, either in the GDM versus healthy group (Table 3). Spearman correlation analysis showed that the total concentration of HMOs in colostrum in the GDM group had a positive correlation with WAZ at 6 months. Moreover, the concentration of LNnT in colostrum and transitional milk was significantly and positively correlated with WAZ at 6 months; LNFP-II had a significant negative correlation on the length and weight for age of infants in all periods. In the healthy group, the concentration of 3′-SL in colostrum was significantly and negatively correlated with WAZ at 3 months. DSLNT in transitional milk showed a negative correlation with WAZ at 3 months (Figure 3).

No differences were observed in the number of bowel movements and stool properties of the infants at 42 days after delivery in the two groups. Regarding disease status, an infant in the healthy group had disease (respiratory disease) within the first 42 days after delivery and was hospitalized; regarding feeding status, two mothers in each group fed their infants by mixed feeding at 42 days postpartum, with a ratio of breast milk to formula feeding ranging from 6:1 to 1:1. Neither disease status nor feeding status was observed to differ between the two groups (Supplementary Table S4).

## 4. Discussion

Breast milk contains a variety of HMO structures, of which so far over 200 were identified. However, the majority of the total HMO concentration was only contributed by the top 10~15 structures [9]. Specifically, 2′-FL, LNDFH-I, LNFP-I, LNFP-II, LNT, 3-FL, 6′-SL, DSLNT, LNnT, Difucosyllactose (DFL), Fucosyldisialyllacto-N-hexaose I (FDS-LNH-I), LNFP-III, 3′-SL, LST c, and Trifucosyllacto-N-hexaose (TF-LNH) appear to constitute the majority of the total HMO components (>75%) of mature milk [9]. A total of 12 of these 15 individual HMOs were measured in this study, as well as 2 individual HMOs of interest to us. The 14 HMOs in this study could represent somewhat the majority of the total level of HMOs.

HMOs fucosylation patterns were determined by secretor and Lewis status. The proportion of different phenotypes varies by race. Many studies have assessed the quantitative variation in HMO between secretor and non-secretor individuals. Surveys in Europe, Asia, and Africa [13] showed that Se^+^Le^+^ mothers were the most predominant group (45–77% of those surveyed); the second predominant group was Se^−^Le^+^ (7–34%), followed by Se^+^Le^−^ (4–28%) and Se^−^Le^−^ (1–26%). Another study reported typical distributions of Se^+^Le^+^, Se^−^Le^+^, Se^+^Le^−^, and Se^−^Le^−^ in the global population as 70%, 20%, 9%, and 1% [9]. In our study, the number of Se^+^Le^+^ phenotype (68.2%) was comparable to the global average.

Most of the HMOs showed a significant temporal trend with decreasing levels with lactation time; however, there were some exceptions, where 2′-FL, 3-FL, and LNFP-II remained stable during lactational period in the GDM and healthy groups, and LNFP-III peaked in mature milk in the GDM group. Studies had shown that 2′-FL was associated with stimulation of brain development, improved cognitive outcomes, and rapid increases in infant weight gain [41]. A significant increase in 3-FL after colostrum was also reported by Wang et al. [17], Plows et al. [41], Gu et al. [42], and Soyyilmaz et al. [9]. However, we did not observe this trend, and 3-FL remained constant in our study. 3-FL and 2′-FL have both been found to bind to norovirus and naturally act as a decoy against norovirus infection. Thus, adequate concentrations of 3-FL and 2′-FL may provide ongoing immune support for growing infants [41]. Moreover an animal experiment by Bhargava et al. [43] showed that LNFP-III could affect glucose homeostasis in a mouse model. The application of immunomodulatory HMO LNFP-III has been shown to improve glucose tolerance and insulin sensitivity by stimulating IL-10 production in macrophages and dendritic cells, reducing white adipose tissue inflammation and sensitizing adipocytes to insulin responses. This may explain the increase in LNFP-III content with lactation time in the GDM group.

After controlling for most of the factors reported in the literature that may affect HMOs, we found that HMOs were significantly different in the healthy and GDM groups in terms of the content of LNnT, LNFP-II, 3′-SL, and DSLNT in the Se^+^Le^+^ group. Compared with studies of the same type, the study by Smilowitz et al. [30] did not find a difference in HMOs in breast milk between the GDM group (n = 8) and the healthy group (n = 16) two weeks after delivery, which may be due to the imbalance in the number of participants between the two groups and the small sample size of participants in the GDM group. Moreover, the authors did not classify the secretory phenotypes. Another research that explored the difference between GDM and healthy donors showed that the concentration of 3′-SL in colostrum of mothers in the secretor GDM group (n = 18) was significantly lower than that of mothers in the healthy group (n = 43) (GDM: 144 ± 161 mg/L versus healthy: 252 ± 181 mg/L, *p* < 0.05), which was the only individual HMO that differed between the two groups [31]. Moreover, not only was the 3′-SL content significantly lower in the GDM group than in the healthy group, but also the total sialylated HMOs content was lower than in the healthy group. This is different from our results, which showed higher 3′-SL and sialylated HMOs in the secretor GDM group than in the healthy group. In order to compare with this study, we screened only Se^+^ phenotype mothers without considering Lewis gene and reached the same conclusion as the Se^+^Le^+^ mothers. The 3′-SL and sialic HMOs in the breast milk of GDM mothers with Se^+^ phenotype were higher than those in the healthy group. Since the authors did not control for other disease conditions (pregnancy-induced hypertension and hypothyroidism), weight, and other factors when analyzing the relationship between GDM and HMO, this may have contributed to the difference in our study results.

The increase in sialylation may occur in patients with diabetes. A recent study found increased sialylation of plasma N-glycans in patients with type 2 diabetes [44]. The authors proposed inflammatory processes as a possible underlying mechanism. Inflammation is associated with altered glycosylation caused by external sialylation of extracellular sialyltransferases [45]. Whether increased sialylation is a general response to inflammation in pregnancy and whether these changes in sialylation have an ameliorating or worsening effect remains to be elucidated. Additionally, increased sialylation may alter the interactions between carbohydrates on N-glycans and lectins, such as selectins or galactose lectins, with potentially profound effects on immune regulation and metabolism [46]. HMOs have been circulating in the bloodstream of pregnant women since at least week 10, through the amniotic fluid and possibly into the placenta via the umbilical cord [47]. 3′-SL is one of the most abundant HMOs in maternal serum, especially in early gestation, when alpha 1–2 focusing has not yet begun [26]. It has been shown that 3′-SL is closely associated with fasting blood glucose concentrations (changes) in early pregnancy, and the higher the 3′-SL concentration, the greater the increase in fasting blood glucose [27]. This may explain the higher total sialylated HMOs and 3′-SL in the GDM group compared to the healthy group. In GDM cases, if treatment allows the normalization of glucose and insulin parameters, it may similarly restore 3′-SL concentrations [46]. However, the sample size of this study was too small to investigate whether good or poor glycemic control might also affect 3′-SL. In a larger cohort of GDM patients receiving insulin or dietary advice, it would be interesting to investigate the potential impact of the respective type of treatment and its effectiveness on maternal 3′-SL.

Gestational diabetes mellitus is a risk factor for the development of serious diseases such as necrotizing enterocolitis (NEC) in newborns. A large retrospective cohort study showed that maternal gestational diabetes mellitus was associated with the incidence of severe neonatal disease compared to mothers without diabetes (OR = 1.16, 95% confidence interval (CI) = 1.04–1.30) [48]. Sialylated HMO is associated with the protective effect of breast milk against pathogenic infections and NEC, especially DSLNT, which is known for its prevention of NEC [46,49] and antibacterial and anti-biofilm activity against group B *Streptococcus* [50]. The higher DSLNT content in the colostrum of the GDM group may help to give more protection under higher risk. In addition to this, one study found a lower abundance of *Bifidobacteria* and *Bacteriodes* in infants delivered by cesarean section, with *Clostridium difficile* predominance [51]. Since DSLNT can reduce pathogenic infections, this may explain the higher levels of DSLNT in maternal HMOs in the healthy group of cesarean section compared to vaginal deliveries in colostrum.

Comparisons between groups revealed that LNFP-II levels were significantly higher in both colostrum and transitional milk of healthy mothers than in the GDM group, and that LNFP-II levels in the GDM group showed a significant negative correlation with infant WAZ and LAZ at each period, and that this effect lasted until 6 months postpartum. Although it is difficult to give a plausible explanation, this unique finding may reveal key HMOs affecting infant growth and development in the GDM group and needs to be further validated in non-secretor lactating mothers and in larger samples. Moreover, for Se^+^Le^+^, maternal age was negatively associated with LNFP-II. This result may be related to age-induced changes in the body. However, the extent of the effect remains uncertain. This is different from the influence factors derived from other studies [17,52].

HMOs are intrinsic components that influence the intestinal microbiota by providing a source of energy for beneficial intestinal bacteria. LNnT has been shown to alter the human intestinal flora [53]. Another study reported that infants fed formula containing 2′-FL and LNnT developed a gut microbiota by an increase in the abundance of beneficial bifidobacteria and a decrease in the abundance of taxa with potentially pathogenic members [54]. The microbiota of GDM offspring showed an abundance of pro-inflammatory taxa. In a comparison of breastfed infants in the GDM and healthy groups, the offspring of GDM mothers showed increased abundance of pro-inflammatory taxa [55]. LNnT was higher in the GDM group than in the healthy group at all three time points, possibly indicating a role for LNnT in maintaining the intestinal flora of the GDM group.

Comparing our study with Alderete et al. [32] and Larsson et al. [34], the similarity is that LNnT is associated with growth; however, unlike our study, Alderete et al. found a negative association with fat mass (FM)%, with each 1 μg/mL increase in LNnT being associated with a 0.03% decrease in body fat (β = −0.03, *p* < 0.01), and Larsson et al. found that the high weight-gain (HW) group LNnT values were lower and FM% was significantly higher in the HW group compared to the healthy weight-gain (NW) group. In addition, a negative correlation between LNnT and FM index at 5 months was also found. Our study showed that LNnT in colostrum and transitional milk showed a significant positive correlation with WAZ at six months in the GDM group. The levels of HMOs in the GDM and healthy groups also showed that LNnT was significantly different between the two groups, and both were higher in the GDM group than in the healthy group. The role of LNnT in regulating intestinal flora in the offspring of GDM may make its effect on infant growth different from that of the offspring of healthy mothers.

In the study conducted by Davis et al. [33], they observed that a higher proportion of LST c in breast milk was associated with lower WAZ of infants and that a higher proportion of 3′-SL contributed positively to WAZ and LAZ; however, our study showed that 3′-SL as well as DSLNT showed a negative association with WAZ at 3 months of age in the healthy group of infants. We also found that the total HMOs content in colostrum showed a positive correlation with the infant’s WAZ at six months postnatal. The observed correlation between HMO concentrations and infant weight/length suggests a potential role of HMO on infant growth and metabolism, which deserves future study.

In addition to this, it has been shown that the concentration of HMOs is also associated with infant disease status [17]. This may be related to the effect of HMOs on the health of the developing immune system and the establishment of the gut microbiota [17]. However, our study did not find this benefit as the majority of infants from both GDM and healthy mothers remained healthy during investigation.

This study has several limitations. First, the small sample size did not allow us to explore the changes of non-secretor mothers. Second, HMOs have been shown to affect intestinal flora, which have not been explored in our study. Third, the literature has shown that the content of HMOs is also affected by diet [31]. Considering the special situation of the GDM group (all the women with GDM controlled their blood sugar through diet and exercise according to the dietary recommendation), our study did not investigate the relationship between diet and HMOs. It is hoped that subsequent studies can take these into account and carry out relevant design and exploration. Despite these limitations, the current study has important advantages. To our knowledge, this is the first study to comprehensively describe and compare the differences in HMOs between healthy and GDM mothers during lactation at three different stages (colostrum, transitional milk, and mature milk). The longitudinal cohort design makes it feasible to assess prospective changes in HMO concentrations throughout the postpartum period in Se^+^Le^+^ mothers.

## 5. Conclusions

This study explored changes in human milk oligosaccharides over time in GDM and healthy lactating mothers and showed that in Se^+^Le^+^ mothers, the total concentration of HMOs was higher in colostrum of GDM mothers. For individual HMOs, the concentration of LNnT, LNFP-II, 3′-SL, and DSLNT were statistically different between the two groups. Those HMOs may be key factors affecting the growth of infants and may be protective under GDM conditions, which needs further investigation.

## Figures and Tables

**Table 1:** Baseline characteristics of subjects in Se^+^Le^+^ group.

		Healthy Group (n = 8)	GDM Group (n = 7)	*p* Value
Mother’s basic information				
Reproductive age (years, $\overset{-}{x}$±s)		31.13 ± 1.89	31.71 ± 2.43	0.606
Gestation (weeks, $\overset{-}{x}$±s)		39.71 ± 0.87	39.71 ± 0.41	0.996
Production (%)	Primiparous	6 (75.0)	6 (85.7)	1
	Multiparous	2 (25.0)	1 (14.3)	
Occupation (%)	Professionals	1 (12.5)	0	1
	Office clerk	7 (87.5)	7 (100)	
Region (%)	Beijing area	1 (12.5)	0	1
	Non-Beijing area	7 (87.5)	7 (100)	
Education level (Maternity) (%)	Undergraduate and below	2 (25.0)	4 (66.7)	0.277
	Postgraduate and above	6 (75.0)	2 (33.3)	
Education level (Spouse) (%)	Undergraduate and below	1 (12.5)	3 (50.0)	0.245
	Postgraduate and above	7 (87.5)	3 (50.0)	
Monthly income per family member (%)	≤15,000	3 (37.5)	4 (66.7)	0.592
	>15,000	5 (62.5)	2 (33.3)	
Premature rupture of membranes (%)	Yes	0	1 (14.3)	0.467
	No	8 (100)	6 (85.7)	
Delivery (%)	Vaginal delivery	6 (75.0)	5 (71.4)	1
	Cesarean delivery	2 (25.0)	2 (28.6)	
Body temperature (°C, median(Q1–Q3))		36.5 (36.3, 36.6)	36.5 (36.2, 36.5)	0.292
Pulse (bpm, median(Q1–Q3))		90 (72, 100)	80 (80, 90)	0.314
Systolic pressure (mmHg, $\overset{-}{x}$±s)		120.00 ± 7.69	119.71 ± 7.54	0.943
Diastolic pressure (mmHg, $\overset{-}{x}$±s)		75.25 ± 7.55	75.14 ± 5.55	0.976
Height (cm, $\overset{-}{x}$±s)		165.63 ± 4.50	161.14 ± 6.12	0.127
Pre-pregnancy weight (kg, $\overset{-}{x}$±s)		55.50 ± 3.96	58.57 ± 5.65	0.240
Current weight (kg, $\overset{-}{x}$±s)		68.75 ± 6.48	68.21 ± 5.16	0.864
Gestational weight gain (kg, $\overset{-}{x}$±s)		13.25 ± 5.18	9.64 ± 4.77	0.186
Allergic diseases (Maternity)	Yes	4 (50.0)	3 (42.9)	1
	No	4 (50.0)	4 (57.1)	
Allergic diseases (Spouse)	Yes	3 (37.5)	3 (42.9)	1
	No	5 (62.5)	4 (57.1)	
Infant’s basic information				
Gender (%)	Male	2 (25.0)	4 (57.1)	0.315
	Female	6 (75.0)	3 (42.9)	
Birth weight (g, $\overset{-}{x}$±s)		3336.25 ± 267.53	3420.00 ± 320.73	0.590
Birth length (cm, $\overset{-}{x}$±s)		49.50 ± 1.07	50.29 ± 1.25	0.212
Birth head circumference (cm, median(Q1–Q3))		34 (33, 35)	34 (34, 34.5)	0.598

**Table 2:** Changes in various types of human milk oligosaccharides (HMOs) over time in Se^+^Le^+^ group.

HMO Types		Healthy Group (n = 8)			GDM Group (n = 7)		
		T1	T2	T3	T1	T2	T3
NN	LNT	50.25 (42.54, 98.76) ^b^	48.85 (30.33, 82.38) ^c^	29.69 (26.53, 39.70) ^bc^	130.69 (87.26, 147.64) ^b^	106.91 (62.44, 119.97) ^c^	59.62 (21.26, 67.26) ^bc^
	LNnT	34.17 (29.75, 42.69 ^b^	17.38 (11.65, 37.01)	15.90 (9.55, 22.49) ^b^	174.68 (34.05, 240.20) ^ab^*	126.62 (27.55, 176.77) ^ac^*	74.15 (17.48, 82.12) ^bc^*
FN	2′-FL	230.65 (173.45, 336.75)	220.44 (175.24, 326.92)	261.97 (202.52, 328.61)	264.78 (250.50, 300.70)	254.42 (233.85, 296.42)	239.60 (218.65, 298.89)
	3-FL	21.89 (5.59, 85.70)	31.76 (7.32, 71.57)	46.48 (11.43, 74.96)	73.91 (55.36, 136.45)	70.08 (56.30, 102.10)	70.23 (61.08, 106.02)
	LNFP-I	117.46 (53.84, 169.65) ^b^	65.25 (45.60, 144.66)	87.79 (16.29, 108.42) ^b^	156.63 (125.22, 176.69) ^ab^	103.76 (83.93, 123.60) ^ac^	47.95 (39.87, 56.68) ^bc^
	LNFP-II	31.97 (10.44, 46.02)	37.33 (17.08, 40.75)	21.59 (14.35, 28.25)	4.93 (4.22, 5.46) *	4.30 (3.30, 20.51) *	6.44 (4.11, 7.03)
	LNFP-III	21.72 (15.79, 29.27) ^a^	17.28 (11.60, 24.36) ^a^	19.66 (11.91, 26.00)	17.09 (7.31, 22.74) ^a^	12.23 (7.28, 20.03) ^ac^	19.19 (14.20, 21.92) ^c^
	LNDFH-I	66.16 (15.20, 87.10) ^b^	55.85 (49.31, 88.98) ^c^	36.76 (22.99, 68.73) ^bc^	34.50 (25.52, 43.83) ^b^	40.68 (28.39, 53.96) ^c^	26.01 (18.08, 31.78) ^bc^
NS	3′-SL	8.63 (4.42, 11.14)	8.09 (3.76, 9.28)	6.50 (3.38, 9.41)	19.48 (8.02, 23.01) ^b^*	19.26 (7.19, 22.43) ^c^	12.99 (7.58, 19.23) ^bc^*
	6′-SL	30.95 (8.23, 38.37) ^b^	21.90 (7.53, 35.54) ^c^	11.19 (1.84, 16.77) ^bc^	33.81 (23.51, 48.74) ^b^	28.08 (15.66, 41.50) ^c^	11.64 (11.34, 21.42) ^bc^
	LST a	8.42 (2.68, 10.76) ^ab^	4.19 (1.34, 5.51) ^ac^	1.77 (0.65, 2.44) ^bc^	7.88 (5.84, 9.77) ^ab^	4.32 (2.90, 6.12) ^ac^	1.60 (1.56, 3.01) ^bc^
	LST b	8.96 (4.75, 11.65) ^b^	7.83 (3.45, 9.95) ^c^	5.63 (2.95, 7.75) ^bc^	12.71 (9.06, 15.94) ^b^	10.44 (6.24, 16.23) ^c^	5.86 (3.83, 7.90) ^b^
	LST c	27.38 (9.05, 3340) ^ab^	10.24 (8.73, 18.64) ^ac^	4.97 (2.60, 7.21) ^bc^	35.01 (29.49, 41.84) ^ab^	15.78 (8.51, 25.13) ^ac^	4.66 (3.11, 8.78) ^bc^
	DSLNT	45.46 (35.14, 57.83) ^b^	48.88 (28.63, 63.46) ^c^	25.96 (16.25, 37.37) ^bc^	89.20 (41.76, 101.94) ^ab^*	85.00 (27.50, 93.61) ^ac^	34.03 (8.84, 56.26) ^bc^

NN, FN, NS represent non-fucosylated non-sialylated HMOs, fucosylated non-sialylated HMOs, and non-fucosylated sialylated HMOs. T1, T2, and T3 represent colostrum (within 7 days), transitional milk (12–14 days), and mature milk (42 days), respectively. ^a^ indicates significantly different HMO concentrations in colostrum and transitional milk (*p* < 0.05); ^b^ indicates significantly different HMO concentrations in colostrum and mature milk (*p* < 0.05); ^c^ indicates significantly different HMO concentrations in transitional milk and mature milk (*p* < 0.05), according to Bonferroni adjustment test for multiple comparisons. * indicates a statistically significant difference compared with the healthy group.

**Table 3:** Effects of different levels of HMOs on infant growth and development in Se^+^Le^+^ group.

	42 Days after Birth		3 Months after Birth		6 Months after Birth	
	Healthy Group	GDM Group	Healthy Group	GDM Group	Healthy Group	GDM Group
Length	56.00 ± 1.47	57.62 ± 1.26	63.90 ± 1.44	64.75 ± 3.10	68.73 ± 1.42	68.50 ± 0.58
Weight	4942.86 ± 479.09	5460.00 ± 1238.65	7587.50 ± 909.50	6962.50 ± 982.66	8536.25 ± 617.32	8530.00 ± 565.33
Head	37.57 ± 0.73	37.75 ± 1.54	41.23 ± 1.24	40.00 ± 0.50	42.89 ± 1.49	43.33 ± 1.04
LAZ	0.99 ± 0.87	0.81 ± 0.89	1.36 ± 0.33	2.03 ± 2.41	1.05 ± 0.63	1.42 ± 0.42
WAZ	1.02 ± 0.81	0.80 ± 1.62	1.65 ± 1.11	1.12 ± 1.64	1.11 ± 0.63	1.32 ± 0.69
BMIZ	0.70 ± 0.71	0.55 ± 2.64	1.23 ± 1.54	0.06 ± 0.73	0.71 ± 0.71	0.75 ± 1.21

## Data Availability

We can’t release the data due to the restrictions of the informed consent.

## Supplementary Materials

The following supporting information can be downloaded at: www.mdpi.com/article/10.3390/nu15061441/s1, Table S1: Baseline characteristics of subjects; Table S2: Blood glucose for mothers and infants with GDM; Table S3: Distribution of the four milk types; Table S4: Other information about the infants in each group within 42 days of discharge from the hospital.
