# Supplementary material for: Human Milk Oligosaccharides Variation in Gestational Diabetes Mellitus Mothers
Source: Nutrients. 2023 Mar 16;15(6):1441. doi: 10.3390/nu15061441 (PMC10059845; doi:10.3390/nu15061441)
Supplement: Supplementary file 1 [file nutrients-15-01441-s001.zip › nutrients-2245465-supplementary.pdf]

Supplementary table S1-Baseline characteristics of subjects

		Healthy Group(N=11)	GDM Group(N=11)	*P* value
**Mother's basic information**				
Reproductive age(years,median(Q1-Q3))		32(30,33)	31(29,33)	0.949
Gestation(weeks,, $\bar{x}\pm s$)		39.818±0.796	39.564±0.609	0.409
Production(%)	Primiparous	9(81.8)	9(81.8)	1
	Multiparous	2(18.2)	2(18.2)	
Occupation(%)	Professionals	3(27.3)	0	0.214
	Office clerk	8(72.7)	11(100)	
Region(%)	Beijing area	2(18.2)	1(13.6)	1
	Non-beijing area	9(81.8)	10(86.4)	
Education level (Maternity) (%)	Undergraduate and below	4(36.4)	6(66.7)	0.370
	Postgraduate and above	7(63.6)	3(33.3)	
Education level (Spouse) (%)	Undergraduate and below	2(18.2)	6(66.7)	0.065
	Postgraduate and above	9(81.8)	3(33.3)	
Monthly income per family member(%)	≤15000	5(45.5)	5(55.6)	1
	＞15000	6(54.5)	4(44.4)	
Premature rupture of membranes(%)	Yes	1(9.1)	2(18.2)	1
	No	10(90.9)	9(81.8)	
Delivery(%)	Vaginal delivery	8(72.7)	8(72.7)	1
	Cesarean delivery	3(27.3)	3(27.3)	
Body temperature(℃, $\bar{x}\pm s$)		36.56±0.32	35.56±3.10	0.286
Pulse(bpm,median(Q1-Q3))		86(70,100)	80(80,80)	1
Systolic pressure(mmHg, $\bar{x}\pm s$)		117.82±9.94	117.55±7.45	0.943
Diastolic pressure(mmHg, $\bar{x}\pm s$)		72.36±8.52	73.09±5.70	0.816
Height(cm, $\bar{x}\pm s$)		164.64±4.43	162.00±6.02	0.256
Pre-pregnancy weight(kg, $\bar{x}\pm s$)		55.00±4.49	58.64±4.88	0.084
Current weight(kg, $\bar{x}\pm s$)		67.86±6.25	68.91±4.75	0.663
Gestational weight gain (kg, $\bar{x}\pm s$)		12.86±4.40	10.27±4.54	0.189
Allergic diseases (Maternity)	Yes	6(54.5)	4(36.4)	0.670
	No	5(45.5)	7(63.6)	
Allergic diseases (Spouse)	Yes	5(45.5)	4(36.4)	1
	No	6(54.5)	7(63.6)	
**Infant's basic information**				
Gender(%)	Male	5(45.5)	5(45.5)	1
	Female	6(54.5)	6(54.5)	
Birth weight(g, $\bar{x}\pm s$)		3340.00±265.14	3498.18±339.55	0.237
Birth length(cm, $\bar{x}\pm s$)		49.64±1.29	50.46±1.13	0.128
Birth head circumference(cm, $\bar{x}\pm s$)		33.80±0.92	34.10±0.84	0.457
Number of bowel movements (infants, 42days)		3(2,3)	3(3,4)	0.493
Stool characteristics (infants)	Slightly shaped stools	7(63.6)	9(90)	0.311
	Watery stools/banana-like	4(36.4)	1(10)	

	stools/soft stools	

Supplementary table 2 – Blood glucose for mothers and infants with GDM

	N	Minimum	Maximum	Mean	Std. Deviation
**GDM Diagnostic Information**					
Gestational age when GDM was diagnosed (week)	11	24.0	26.5	25.26	0.68
OGTT fasting glucose at 24-28 weeks preganant (mmol/L)	11	4.4	5.3	5.14	0.28
OGTT 1-hour blood glucose at 24-28 weeks preganant (mmol/L)	4	7.4	11.1	9.83	1.67
OGTT 2-hour blood glucose at 24-28 weeks preganant (mmol/L)	4	6.6	9.3	8.23	1.24
Infant's blood sugar at birth (mmol/L)	9	2.30	6.60	3.81	1.52
**Maternity checkup blood glucose level**					
Fasting at 30 weeks pregnant (mmol/L)	10	4.56	5.76	5.06	0.35
Fasting at 32 weeks preganant (mmol/L)	10	4.42	6.00	4.98	0.42
Fasting at 34 weeks preganant (mmol/L)	10	3.85	5.70	5.08	0.56
Fasting at 36 weeks preganant (mmol/L)	9	3.81	5.33	4.73	0.48
Fasting at 38 weeks preganant (mmol/L)	5	4.31	5.50	5.03	0.45
2-hour postprandial at 30 weeks preganant (mmol/L)	10	4.32	6.96	5.69	0.92
2-hour postprandial at 32 weeks preganant(mmol/L)	10	4.57	9.10	6.18	1.37
2-hour postprandial at 34 weeks preganant (mmol/L)	10	4.71	7.44	6.00	0.84
2-hour postprandial at 36 weeks preganant (mmol/L)	8	4.75	7.76	6.03	0.90
2-hour postprandial at 38 weeks preganant (mmol/L)	5	5.61	6.69	6.30	0.45

Supplementary table 3-Distribution of the four milk types

Phenotype	Total	Healthy Group	GDM group
$Se^{+}Le^{+}$ (%)	**15 (68.2)**	**8 (72.7)**	**7 (63.6)**
$Se^{+}Le^{-}$ (%)	**0**	**0**	**0**
$Se^{-}Le^{+}$ (%)	**7 (31.8)**	**3 (27.3)**	**4 (36.4)**
$Se^{-}Le^{-}$ (%)	**0**	**0**	**0**

The data is presented in n (%)

Supplementary table 4 - Other information about the infants in each group within 42 days of discharge from the hospital

		Healthy Group	GDM Group	*P* value
Number of bowel movements (times,median(Q1-Q3))		1(1,2)	1(1,1)	0.180
Stool characteristics (%)	Slightly shaped stools	5(62.5)	6(85.7)	0.338
	Watery stools/banana-like stools/soft stools	3(37.5)	1(14.3)	
Whether the infant was sick within 42 days of discharge from the hospital (%)	Yes	1(12.5)	0	0.533
	No	7(87.5)	7(100.0)	
Whether the infant was hospitalized within 42 days of discharge (%)	Yes	1(12.5)	0	0.533
	No	7(87.5)	7(100.0)	
Feeding status (%)	Breast feeding	6(75.0)	5(71.4)	1
	Mixed feeding	2(25.0)	2(28.6)	
